# Obese rats intervened with *Rhizoma coptidis* revealed differential gene expression and microbiota by serum metabolomics

**DOI:** 10.1186/s12906-021-03382-3

**Published:** 2021-08-11

**Authors:** Yanhua Ji, Kexin Luo, Jiri Mutu Zhang, Peng Ni, Wangping Xiong, Xiaoquan Luo, Guoliang Xu, Hongning Liu, Zhijun Zeng

**Affiliations:** 1 Jiangxi Province Key Laboratory of TCM Etiopathogenisis, Research Center for Differention and Development of TCM Basic Theory, University of Jiangxi TCM, Nanchang, Jiangxi 330006 P. R. China; 2 Laboratory Animal Science and Technology Center, University of Jiangxi TCM, Nanchang, Jiangxi 330006 P. R. China; 3 School of Computer, University of Jiangxi TCM, Nanchang, Jiangxi 330006 P. R. China; 4 Jiangxi Key Lab of Pharmacology of TCM, University of Jiangxi TCM, Nanchang, Jiangxi 330006 P. R. China

**Keywords:** Metabolomics, Obesity, Circadian rhythm, Gut microbiome, *Rhizoma coptidis*

## Abstract

**Background:**

Integrating systems biology is an approach for investigating metabolic diseases in humans. However, few studies use this approach to investigate the mechanism by which *Rhizoma coptidis* (RC) reduces the effect of lipids and glucose on high-fat induced obesity in rats.

**Methods:**

Twenty-four specific pathogen-free (SPF) male Sprague–Dawley rats (80 ± 10 g) were used in this study. Serum metabolomics were detected by ultra-high-performance liquid chromatography coupled with quadrupole-time-of-flight tandem mass spectrometry. Liver tissue and cecum feces were used for RNA-Seq technology and 16S rRNA gene sequencing, respectively.

**Results:**

We identified nine potential biomarkers, which are differential metabolites in the Control, Model and RC groups, including linoleic acid, eicosapentaenoic acid, arachidonic acid, stearic acid, and L-Alloisoleucine (*p* < 0.01). The liver tissue gene expression profile indicated the circadian rhythm pathway was significantly affected by RC (Q ≤ 0.05). A total of 149 and 39 operational taxonomic units (OTUs), which were highly associated with biochemical indicators and potential biomarkers in the cecum samples (FDR ≤ 0.05), respectively, were identified.

**Conclusion:**

This work provides information to better understand the mechanism of the effect of RC intervention on hyperlipidemia and hypoglycemic effects in obese rats. The present study demonstrates that integrating systems biology may be a powerful tool to reveal the complexity of metabolic diseases in rats intervened by traditional Chinese medicine.

**Supplementary Information:**

The online version contains supplementary material available at 10.1186/s12906-021-03382-3.

## Background

A Western diet (high fat) is increasingly popular in China [1], resulting in obesity and diabetes [2], cardiovascular diseases and atherosclerosis. However, the mechanisms underlying this diet-induced obesity remain unclear. Human symbiotic gut microbiota has recently been linked to obesity-related diseases [3–5]. A growing body of studies have suggested that gut microbiota and the metabolites of intestinal microbiota play an important role in the development of obesity-associated diseases [6–10]. Therefore, how to regulate the gut microbiota of obese individuals by diet [11, 12], drugs, or probiotics and prebiotics [13], attracts scientific attention.

With nearly 2000 years of clinical use, *Rhizoma coptidis* (RC) is still used today in traditional Chinese medicine (TCM). RC is widely used for treatment of obesity and its complications [14].

RC possesses common clinical therapeutic effects, including antihyperglycemia and antihyperlipidemia [14, 15]. The main pharmacological active ingredients in RC include berberine, epiberberine, coptisine and palmatine, all of which are alkaloids. Modern pharmacological studies show that the alkaloids from RC can reduce glucose, lipids, and inflammatory cytokines levels [16–19]. Jiang et al. (2004) showed that berberine is a novel, cholesterol-lowering drug that is different from statins [20]. Because RC alkaloids are mainly distributed in the liver and intestine of rats [21], the modulation of gut microbiota and its influence on lipid metabolic pathways are very important aspects of the effects of RC alkaloids on glucose and lipids. A study of B6 mice found that alkaloids could alleviate hyperlipidemia by modulating gut microbiota and bile acid pathways [22]. Additionally, the berberine of RC recently demonstrated effects on preventing high-fat-diet-induced obesity and insulin resistance by altering gut microbiota [23]. The above findings highlight the critical role of RC alkaloids in hyperlipidemia treatment in an obesity model. In addition, the clinical application of alkaloids was found to be more suitable for the obesity stage of prediabetes [24, 25]. Despite these advances, we know very little about the mechanism by which hyperlipidemia and hypoglycemic effects are affected by RC intervention in an obesity model.

Integrating systems biology reveals an efficient method for investigating the etiology of complex metabolic diseases, particularly the application of metabolomics in gut microbiota research [26]. The bacteria producing short-chain fatty acids (SCFAs) are regulated by berberine in Wistar rats [27].

In the present study, we performed an untargeted metabolomics analysis, RNA sequencing and 16S rRNA gene sequencing of gut bacteria to elucidate the mechanism by which RC mitigates the lipids and glucose effect on high-fat-induced obesity in rats.

## Methods

### Materials and reagents

We used HPLC-grade acetonitrile and methanol purchased from Merck (Germany). HPLC-grade formic acid was obtained from Dikma (USA). According to the determination method recorded in the China pharmacopoeia (2020 edition), we prepared the RC decoction and performed quality control (Supplementary Fig. 1 in Supplementary Information). Detailed information for RC preparation is provided in the RC decoction standards within the Supplementary Materials and Methods section in the Supplementary Information. The QIAamp Stool DNA kits (Germany) was used to extract the total DNA of the gut microbiota from cecum collected from the rats killed. The 16S rRNA gene V4 region amplification, sequencing, and bioinformatics analysis are described in detail in the Supplementary Materials and Methods section in the Supplementary Information. The total RNA in the liver was determined using the TRIzol method, details of the statistical analysis are provided in the Supplementary Materials and Methods section in the Supplementary Information.

### Animal experiments

All experiments involving rats were performed with protocols approved by the Animal Ethics Committee of Jiangxi University of TCM. We purchased 24 specific pathogen-free (SPF) male Sprague–Dawley rats (80 ± 10 g) from the Hunan Slac Jynda Laboratory Animal Company (Hunan, China). After 1 week of adaptive feeding by free diet and drinking in a barrier system in the laboratory animal science and technology center of Jiangxi University of TCM, all 24 rats were randomly divided into two groups: (1) Control group (*n* = 8), raised with normal chow diet (containing 10% fat by energy; Supplementary Table 1 in Supplementary Information); (2) Model group (*n* = 16), raised with high-fat diet (containing 60% fat by energy; Supplementary Table 1 in Supplementary Information). After 8 weeks of breeding, the Model group rats were randomly separated into two groups: (1) Model group (*n* = 8), continually fed with high-fat diet; and (2) Model + RC group (*n* = 8), continuously fed with high-fat diet and additional stomach filling with the RC raw medical materials by 0.05 g/kg body weight once daily. Four weeks later (after drug intervention), all animals were euthanized by cervical dislocation after fasting but with free access to water overnight. The liver and feces were excised and collected, weighed, and frozen in liquid nitrogen immediately for further analysis. Weight and length were measured every week, and blood was collected every 2 weeks throughout the experiment. All animals were raised in the same environment and treated in the same way.

### Metabolomic analysis

All blood samples were centrifuged at 4 °C (1500 rpm, 15 min) to isolate serum. In order to obtain small substances with a molecular weight less than 1000 Da, we employed conventional methanol precipitation protein methods [28]. The water ACQUITY UPLC™ *I*-Class Xevo G2-XS QTOF system (USA) was used to detect the metabolic profile in ESI positive-ion modes. The chromatographic conditions were carried out on Waters BEH-C_18_ columns (2.1 × 50 mm, 1.7 μm) to separate the serum blood samples. Optimized UPLC and MS settings for analysis in the only ESI + mode is shown in Supplementary Table 2 in the Supplementary Information.

### Data processing and statistical analyses

Metabolomics data were acquired from the Masslynx software (USA) and the raw data were analyzed by Progenesis QI software (Version 2.3, UK). First, peak picking was aligned and retention time (RT) was calibrated. Then, all the data were imported into the EZinfo software (Version 3.0, Swit) for multidimensional statistical analysis after experimental design setup, including principal component analysis (PCA) and partial least-squares-discriminant analysis (PLS-DA). The quality control parameters for m/z satisfied the minimum coefficient of variation (CV) < 30%, ANOVA *p* value < 0.05, max fold change ≥ 2.0 and VIP > 1 and were chosen for potential biomarkers. The potential biomarkers were further identified by METLIN, KEGG, and HMDB databases as well as via Ingenuity Pathway Analysis (IPA) software [29]. Finally, the differences between the verified potential biomarkers between the Model and Model + RC groups were performed by one-way analysis of variance using the software program R (http://www.r-project.org/).

## Results

### RC reduces glucose in high-fat diet-induced obese rats

After 8 weeks of high-fat diet breeding, we found the weight, length, and lee’s index (LI) of the Model group (obesity) to be higher than that of the Control group raised on a normal chow diet (Supplementary Table 3 in Supplementary Information). In addition, the levels of total cholesterol (TC), glucose (GLU) and homeostasis model assessment of insulin resistance (HOMA-IR) of the Model group were also higher than those of the Control group (Supplementary Table 3 in Supplementary Information). However, after 4 weeks of oral administration of RC, only GLU and HOMA-IR were significantly reduced when compared with the Model group (Supplementary Table 4 in Supplementary Information). Meanwhile, the level of TC was still higher than that of the Control group. In addition, we analyzed the amount of food consumed after ingestion of the RC decoction. Compared with the Model group, there was no difference in the average amount of food consumed from the RC group (*p* > 0.05; Supplementary Table 5 in Supplementary Information).

### Serum metabolic profiles and candidate biomarkers

**Fig. 1:**
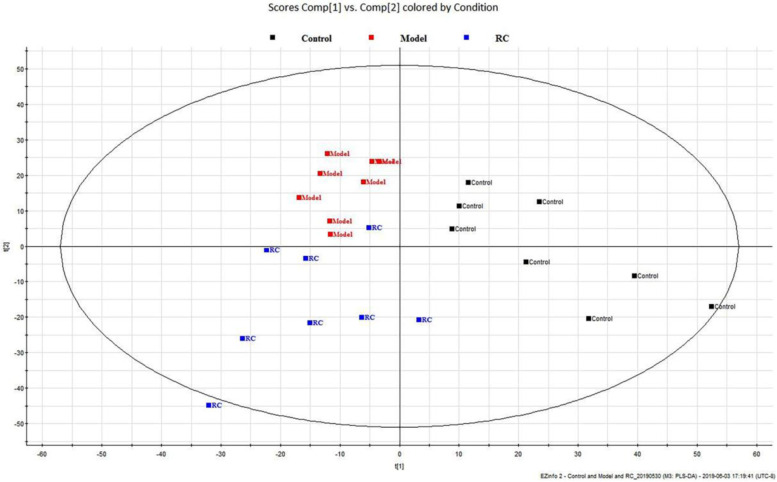
PLS-DA score plots derived from the UHPLC-Q-TOF spectra of the serum in positive mode

**Fig. 2:**
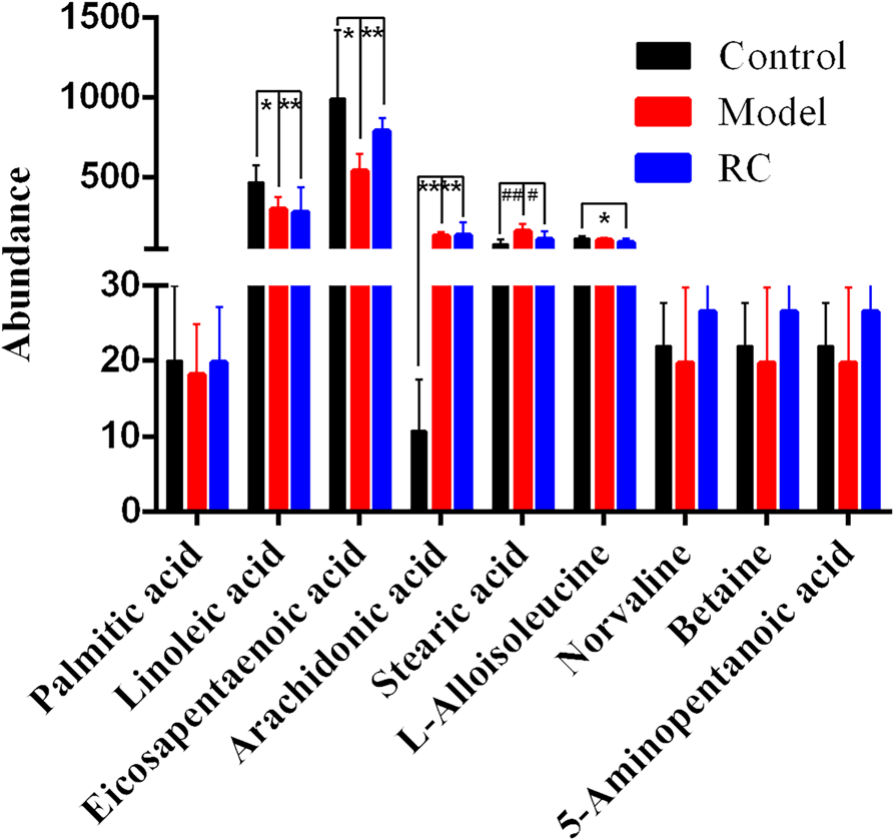
Comparison of the abundance of potential biomarkers in serum from the Control, Model and RC groups. The potential biomarkers are palmitic acid, linoleic acid, eicosapentaenoic acid, arachidonic acid, stearic acid, L-alloisoleucine, norvaline, betaine, and 5-aminopentanoic acid. ^*^p < 0.05, ^**^p < 0.01, ^#^*p* < 0.05, ^##^*p* < 0.01, ^*^ and ^#^ compared between the Control group and Model group, respectively

The total ion chromatograms (TIC) and basic peak intensity (BPI) of serum samples in the positive-ion mode are shown in Supplementary Fig. 2 in Supplementary Information. UHPLC-Q-TOF–MS in the positive MSE model and unadjusted continued data was used to detect small endogenous metabolites in the serum. The PLS-DA results of the Control, Model, and RC groups are shown in Fig. 1. There is a slight separation between the Control and Model groups. Furthermore, potential biomarkers were identified by Progenesis MetaScope of the Progenesis QI software based on accurate mass measurements via UHPLC-TOF–MS. The ion *m/z* 556.2771 of leucine encephalin was used to illustrate the process of biomarker identification. The software adopted an automatic search method with retention time correction and peak alignment. The detailed validation protocol was performed according to Zhang’s methods [28] (Supplementary Fig. 3 in Supplementary Information). The process of searching for potential biomarkers and pathways was as follows: (1) The differential metabolites between the Control and Model groups were defined as palmitic acid, linoleic acid, eicosapentaenoic acid, arachidonic acid and stearic acid, and the associated pathways were from the biosynthesis of unsaturated fatty acids, free fatty acid receptors, α-linolenic acid, linoleic acid metabolism, and the circadian clock (Supplementary Table 6 in Supplementary Information). (2) According to the comparison between the Model and RC groups, in addition to the differential metabolites identified between the Control and Model, branched chain amino acids (norvaline, L-alloisoleucine), betaine and 5-aminopentanoic acid were also found to be differential metabolites between the Model and RC groups (Supplementary Table 7 in Supplementary Information). (3) To further compare the differences among the three groups, we used the Bartlett test of homogeneity of variances in combination with a one-way ANOVA to estimate the p-values, as shown in Fig. 2. Compared to the Control group, the levels of linoleic acid and eicosapentaenoic acid were significantly decreased (*p* < 0.01) in the serum samples of the obese rats raised with a high-fat diet. Although the RC group did not show a reversal of this trend, the levels of eicosapentaenoic acid tended to rise.

### Differential gene expression of liver tissue

**Fig. 3:**
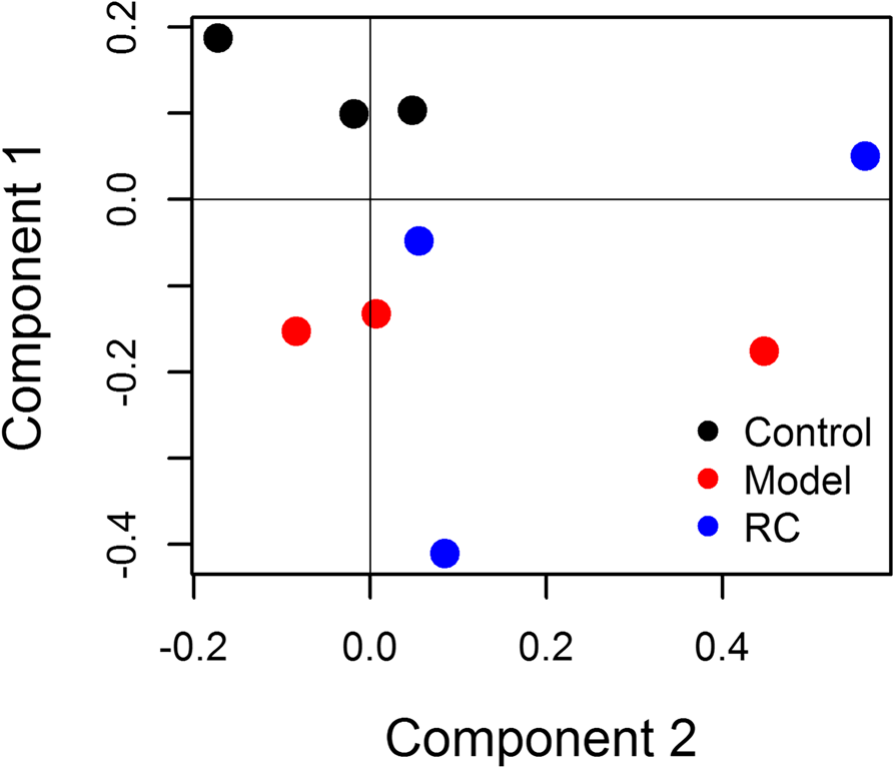
PCA scores plots derived from the transcriptional profiling of the liver tissue

To further explore the changes of related genes in serum metabolites caused by glucose-lowering as well as lipid invariability affected by RC, we carried out RNA-Seq technology of the liver tissue (Supplementary Materials and Methods in Supplementary Information). Since most drugs are eliminated from the body by hepatic metabolism, we sequenced the total RNA in the liver tissue [30, 31]. The PCA scoring diagram helped to differentiate among the three groups. Compared with the Model group and the Control group, the RC group profile displayed an apparent returning trend (Fig. 3). Hence, the identified differentially expressed genes could contribute to the hypoglycemic effect mechanism of RC in high-fat-diet-induced obese rats.

**Fig. 4:**
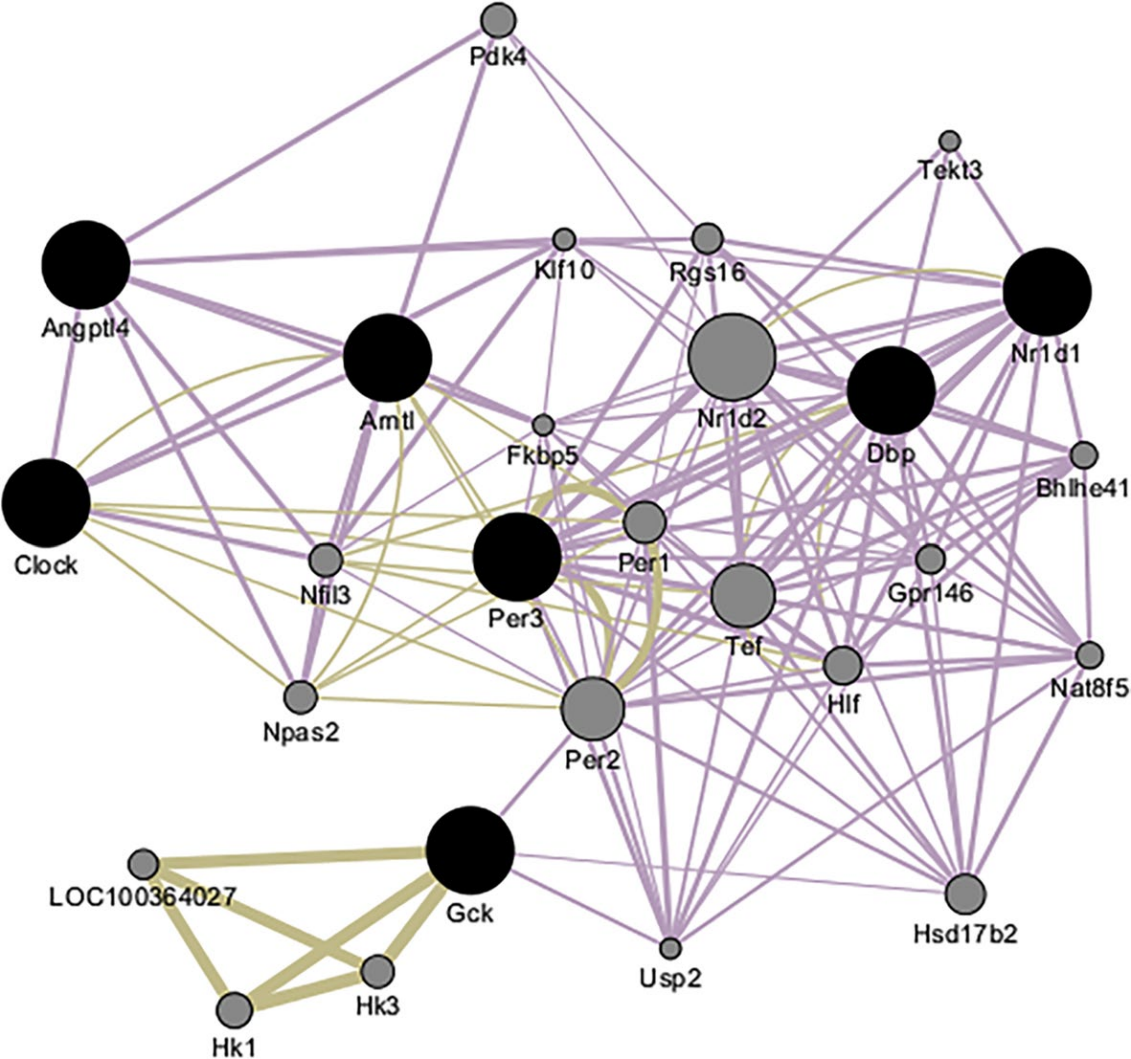
The network of the *Arntl* gene of the circadian rhythm pathway

In order to understand the relationship between the differentially expressed genes, we constructed network relationships [32]. Interestingly, the circadian rhythm pathway was especially affected by the RC, in which priority was given to the *Arntl* gene (Fig. 4 and Supplementary Fig. 4 in Supplementary Information). Compared with the Control group, the expression levels of all seven candidate genes in the Model group were significantly decreased. However, compared to the Model group, the expression levels of these genes in the RC group were significantly increased and were close to, or equal to, the levels in the Control group.

### Differentiated microbiota with biochemical indicators and metabolomic biomarkers

Further studies showed that gut microbes were associated with obesity and produced large amounts of metabolites [33, 34]. To further verify the relationship between serum metabolites and biorhythm genes under the hypoglycemic effect of RC, we executed 16S rRNA gene sequencing of gut bacteria of cecum. It has been reported that cecum luminal samples are more useful for investigation of fatness-associated microbes than stool samples [35]. Hence, we used the cecum sample to study the gut microbiota. We used the association studies with a two-part model to screen out the biomarkers and biochemical indicators related operational taxonomic units (OTUs) (Supplementary Materials and Methods in Supplementary Information) [36]. Corrections were first made for the body weight and length values, and then the residuals were used for association. In the biochemical indicator results, we identified a total of 149 significant associations for 88 shared OTUs at FDR ≤ 0.05 for TC and high-density lipoprotein cholesterol (HDL-C) (Supplementary Fig. 5 in Supplementary Information). These OTUs were mainly annotated to *Clostridium viride*, *Butyricicoccus pullicaecorum*, *Lachnospiracea* and *Ruminococcaceae*.

**Fig. 5:**
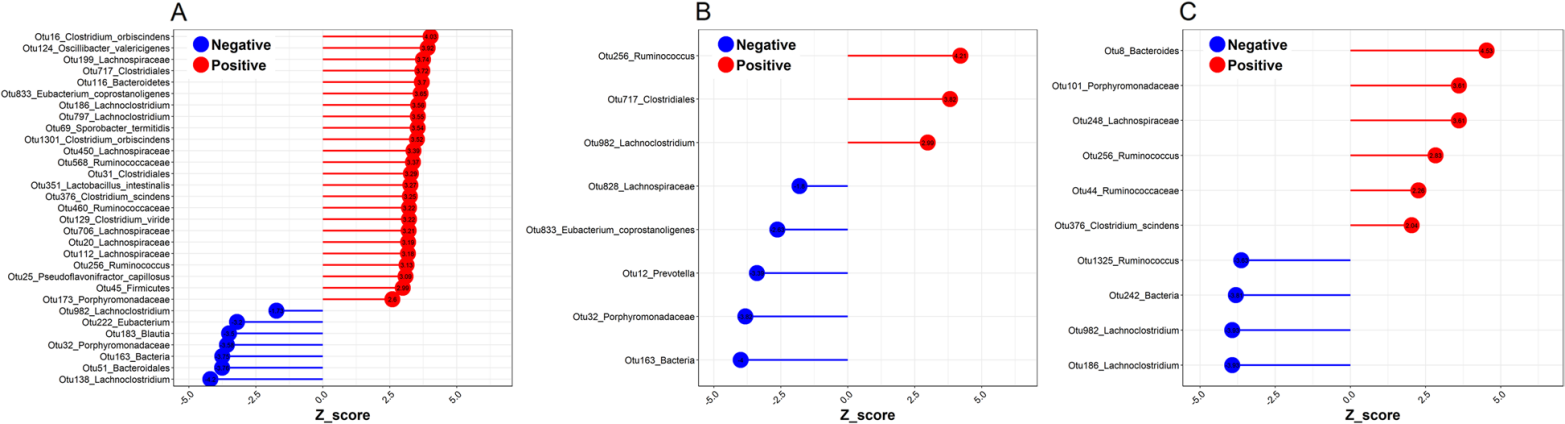
The relationship between the endogenous metabolites and gut microbiota. **A** The results of linoleic acid. **B** The results of eicosapentaenoic acid. **C** The results of arachidonic acid. Thirty-nine OTUs were significantly associated with linoleic acid, eicosapentaenoic acid and arachidonic acid at FDR ≤ 0.05. Otu982 and Otu256 were shared among the three metabolites

With respect to the potential biomarkers, we identified 39 OTUs that were significantly associated with linoleic acid, eicosapentaenoic acid and arachidonic acid at FDR ≤ 0.05 (Fig. 5). These OTUs were annotated to the three dominant Phyla (Firmicutes, Bacteroidetes and Proteobacteria). Of the 39 metabolite-associated OTUs, two (Otu982 and Otu256) were shared among the three metabolites. These two OTUs were annotated to *Lachnoclostridium* and *Ruminococcus*, respectively. *Lachnoclostridium* showed a negative association with both linoleic acid and arachidonic acid (*P* = 1.00E – 04 and 3.77E – 05, respectively) and a positive association with eicosapentaenoic acid (*P* = 3.91E – 05). *Ruminococcus* was positively associated with linoleic acid, arachidonic acid and eicosapentaenoic acid (*P* = 1.00E – 04, 3.77E – 05 and 2.58E – 05, respectively) (Fig. 5). The OTU376 associated with TC, HDL-C, linoleic acid and arachidonic acid was classified as *Clostridium scindens*. *Clostridium scindens* showed strong negative associations with the biochemical indicators and positive associations with potential biomarkers. As for other biochemical indicators and potential biomarkers, we did not identify any significant associations at the OTU level.

## Discussion

The present study aimed to elucidate the mechanism by which RC mitigates the glucose effect on high-fat-induced obesity in rats. The results of the serum biochemical indicators showed that RC could effectively lower blood glucose, which is consistent with clinical observations [37]; however, RC did not reduce the serum lipids. We noticed that the observed results were independent of the amount of food ingested. The reason for this result may be the alkaloids, which are the main components that have inhibitory activities of α-glucosidase in RC extract [38, 39].

We also evaluated the serum metabolic profiles and found that the differential metabolites were associated with the metabolites of unsaturated fatty acids and SCFAs. Linoleic acid can reduce triglyceride storage [40] and lead to a reduction in total serum cholesterol [41], diminishing glucose uptake and utilization [40]. Arachidonic acid, as a metabolite of linoleic acid, can regulate cholesterol metabolism [42]. Eicosapentaenoic acid, which belongs to a different family of polyunsaturated fatty acids, can also alleviate and/or prevent obesity [43]. Eicosapentaenoic acid inhibited hyperglycemia through a potent antioxidant mechanism [44]. These preliminary studies showed that linoleic acid and arachidonic acid both have a good effect on lipid regulation in obesity, while eicosapentaenoic acid has a good effect on hypoglycemia. This is consistent with the results observed after administration of the RC in this study. Only the level of eicosapentaenoic acid increased in the RC group. Results of these potential metabolites support the data regarding the serum biochemical indicators. After RC treatment, the level of betaine increased compared to the Model and Control groups. Additionally, a recent study suggested that supplementation of a native betaine source increased propionic acid production [45]. In addition, these differential metabolites were produced by intestinal microbiota and confirmed the effect of RC on gut microbiota [45–47].

We found that the difference of gene enrichment was mostly caused by sugar and lipid metabolism related genes and their pathways, except for the *Dbp* gene. However, one study showed that the *Dbp* gene binds to saturated and unsaturated fatty acids [48]. This suggested that the RC could have a great influence on the starting position of the loop in the biorhythm pathway [49]. The above results verified that blood glucose and HOMA-IR were affected by serum metabolite enrichment of the biological clock pathway. Circadian rhythm changes could have a profound effect on human health, and up to 15% of human genes have been regulated by patterns of circadian rhythm. Nearly 50% of genes involved in metabolism pathways found in the liver were under the influence of this rhythm [50].

Gut microbes play an important role in obesity. Interestingly, we found the OTUs that were associated with TC and HDL-C produced SCFAs such as *Clostridium viride*, *Butyricicoccus pullicaecorum* [51], *Lachnospiracea* and *Ruminococcaceae* [52, 53]. Regarding the differential metabolites we screened, as well as the results related to OTU, we found that these OTUs, which mainly were annotated as *Lachnospiracea* and *Ruminococcaceae* [54], were also SCFAs producing bacteria. These bacteria helped increase fecal SCFAs concentrations, promote energy intake from fiber, inhibit opportunistic pathogens, and protect the hosts against inflammation and colonic diseases [55].

At the OTU level, the biochemical indicators and differential metabolites associated OTUs were annotated as *Clostridium scindens*, which converted glucocorticoids into androgens by cleaving the carbon–carbon bond of 17-hydroxylated corticoids at C17-C20 [56]. According to studies of humans and rats [57], *Clostridium scindens* is involved in the synthesis of bile acid and may inhibit the growth of *Clostridium difficile*.

A recent study by De Preter (2015) described systems biology as an integrative research strategy that studies the interactions between DNA, mRNA, protein, and metabolite level in an organism [58]. Based on the integrating systems biology research strategy, Mardinoglu et al. (2018) used a multi-omics approach to characterize the resulting alterations in metabolism, transcript profiling of liver biopsies, and the gut microbiota [59]. In future studies, integrating systems biology would greatly promote glycolipids mechanism research on the complexity of metabolic diseases intervened by traditional Chinese medicine. Furthermore, the results from this study provide important insight, such as isolation of the causative microbes for RC, and mitigation of the glucose effect on high-fat-induced obesity in rats, that provide basic information for regulating the gut microbes to reduce the occurrence of obesity.

## Conclusion

Collectively, by systematically combining untargeted metabolomics, RNA-Seq sequencing and 16S rRNA gene analysis techniques, which proved to be an efficient and robust way of identifying potential biomarkers from many metabolites, nine potential biomarkers were found that showed significant associations with the circadian rhythm pathway. Furthermore, a major gene (*Arntl* gene) was affected by RC intervention. The potential biomarkers and biochemical indicators associated with gut microbiota were mainly affected by the SCFAs producing bacteria. These results suggested that the circadian rhythm pathway may play an important role in the metabolites of serum from obese rats as well as the gut microbiota composition. We established a simple research strategy for integrating systems biology and provided information to better understand the mechanism of the effect of RC intervention on the hypoglycemic effect in obese rats.

## Supplementary Information

**Additional file 1:** Supplementary Materials and Methods. **Supplementary Table 1.** The diet composition on normal chow diet and high-fat diet. **Supplementary Table 2.** UPLC elution gradient for optimized UPLC-MS methods, ESI+. A is 99.9/0.1 water/formic acid and B is 99.9/0.1 acetonitrile/formic acid. **Supplementary Table 3.** Pre-administration: the of results the apparent indexes and serum biochemical indexes ($\overline{x} \pm s$). **Supplementary Table 4.** After dosing: the of results the apparent indexes and serum biochemical indexes ($\overline{x} \pm s$). **Supplementary Table 5.** After dosing: the of results amount of food ingested. **Supplementary Table 6.** The differences metabolites and association pathways of the Control and Model groups. **Supplementary Table 7.** The differences metabolites and association pathways of the RC and Model groups. **Supplementary Figure 1.** HPLC fingerprint of the RC decoction. (A) HPLC-UV chromatogram of Batch A. (B) HPLC-UV chromatogram of Batch B. Peaks were detected at 345nm (1, coptisine; 2, palmatine; 3, berberine hydrochloride). **Supplementary Figure 2.** The chromatograms of the serum sample of the rats in positive mode. (A) Typical TIC chromatogram obtained from the same serum sample of the rats with positive mode. (B) Typical BPI chromatogram obtained from the same serum sample of the rats with positive mode. **Supplementary Figure 3.** Identification of a potential marker. MS/MS spectrum; the collision energy was 20eV~30eV. **Supplementary Figure 4.** The pathway enrichment of differentially expressed genes of the liver tissue. **Supplementary Figure 5.** The relationship between the biochemical indicators and gut microbiota. (A) The results of TC. (B) The results of HDL-C.

## Data Availability

Serum metabolism, fecal microbiota, liver tissue expression profiles and correlation analysis data are available from the corresponding author on reasonable request.

## Abbreviations

TCM: Traditional Chinese medicine
RC: *Rhizoma coptidis*
SPF: Specific pathogen-free
OTUs: Operational taxonomic units
FDR: False discovery rate
RT: Retention time
PCA: Principal component analysis
PLS-DA: Partial least-squares-discriminant analysis
CV: Coefficient of variation
IPA: Ingenuity Pathway Analysis
LI: Lee’s index
TC: Total cholesterol
GLU: Glucose
HOMA-IR: Homeostasis model assessment of insulin resistance
TG: Triglycerides
HDL-C: High-density lipoprotein cholesterol
LDL-C: Low-density lipoprotein cholesterol
TIC: Total ion chromatograms
BPI: Basic peak intensity
SCFAs: Short-chain fatty acids
HPLC–UV: Liquid chromatography with ultraviolet detector
RDP: Ribosomal Database Project
