# Supplementary material for: Obese rats intervened with Rhizoma coptidis revealed differential gene expression and microbiota by serum metabolomics
Source: BMC Complement Med Ther. 2021 Aug 11;21:208. doi: 10.1186/s12906-021-03382-3 (PMC8359625; doi:10.1186/s12906-021-03382-3)
Supplement: Supplementary file 1 — Additional file 1: Supplementary Materials and Methods. Supplementary Table 1. The diet composition on normal chow diet and high-fat diet. Supplementary Table 2. UPLC elution gradient for optimized UPLC-MS methods, ESI+. A is 99.9/0.1 water/formic acid and B is 99.9/0.1 acetonitrile/formic acid. Supplementary Table 3. Pre-administration: the of results the apparent indexes and serum biochemical indexes ($\overline{x} \pm s$x¯±s). Supplementary Table 4. After dosing: the of results the apparent indexes and serum biochemical indexes ($\overline{x} \pm s$x¯±s). Supplementary Table 5. After dosing: the of results amount of food ingested. Supplementary Table 6. The differences metabolites and association pathways of the Control and Model groups. Supplementary Table 7. The differences metabolites and association pathways of the RC and Model groups. Supplementary Figure 1. HPLC fingerprint of the RC decoction. (A) HPLC-UV chromatogram of Batch A. (B) HPLC-UV chromatogram of Batch B. Peaks were detected at 345nm (1, coptisine; 2, palmatine; 3, berberine hydrochloride). Supplementary Figure 2. The chromatograms of the serum sample of the rats in positive mode. (A) Typical TIC chromatogram obtained from the same serum sample of the rats with positive mode. (B) Typical BPI chromatogram obtained from the same serum sample of the rats with positive mode. Supplementary Figure 3. Identification of a potential marker. MS/MS spectrum; the collision energy was 20eV~30eV. Supplementary Figure 4. The pathwa [file 12906_2021_3382_MOESM1_ESM.zip › Supplementary InformationR5.docx]

**Supplementary Information**

**Files in this data supplement**

Supplementary Materials and Methods

Supplementary Table 1

Supplementary Table 2

Supplementary Table 3

Supplementary Table 4

Supplementary Table 5

Supplementary Table 6

Supplementary Table 7

Supplementary Figure 1

Supplementary Figure 2

Supplementary Figure 3

Supplementary Figure 4

Supplementary Figure 5

**Supplementary Materials and Methods**

**Standards of the RC** **decoction**

The *Rhizoma coptidis* (RC) used in this study was purchased from Jiangxi Jiangzhong traditional Herbal Pieces Factory (batch number: 150128) and identified by Professor Yong Liu, Department of Chinese medicinal resources, School of Pharmacy, Jiangxi University of Traditional Chinese Medicine, China. The following was the preparation of the decoction of RC in detail.

Firstly, added 0.5-kilogram of the RC into the boiling pot. Secondly, added 10 times of weight of water and soaked for 1 hour. Thirdly, boiled the herbs for 50 minutes and filtrated the decoction using filters (diameter smaller than 0.5 mm). Fourthly, concentrated the decoction into crude drugs as 1g/mL. Fifthly, the concentrated RC decoction were stored at 80°C until utilized. Then, according to the determination method recorded in the China Pharmacopoeia (2020 edition), we tested the main components from RC decoction. In a word, two batches of RC decoction were analyzed by high performance liquid chromatography with ultraviolet detector (HPLC-UV), proper number of samples were weighed and extracted with 10 mL of methanol in a water bath at 60°C for 20 min, followed by ultra-sonication for 30 min. After centrifugation, the supernatant was filtrated through a 0.22μm membrane filter, and then injected in a volume of 10μL in HPLC. By HPLC fingerprint analysis, the contents of coptisine, palmatine and berberine hydrochloride in the extracts were detected, respectively. The results showed that the RC decoction was conformed to meet the China Pharmacopoeia standard (Committee, 2020, Supplementary Figure 1, the structural formula of the chemicals was cited from <http://www.chemspider.com/>).

**16S rRNA gene V4 region amplification and sequencing and bioinformatics analysis**

1. 16S rRNA gene V4 region amplification and sequencing

Firstly, we tested the quality of all extracted DNA samples, then all the qualified DNA from each sample was used as the template to expand the V4 region of 16S rRNA genes. The forward and reverse primer were 515F: GTGCCAGCMGCCGCGGTAA and 806R: GGACTACHVGGGTWTCTAAT, respectively. We converted the jagged ends of DNA fragments of the PCR products into blunt ends by using T4 DNA polymerase, Klenow Fragment and T4 Polynucleotide Kinase. Then, added an 'A' base to each 3' end to make it easier to add adapters. After all that, the short fragments would be removed by Ampure beads. Finally, only the qualified library could be used for sequencing. The bioinformatics analysis would be carried on with the qualified sequencing data.

2）bioinformatics analysis

To get the high-quality sequences, all the raw data were filtered [1]. Then paired-ends with overlap were merged to tags, which were clustered to OTU at 97% sequence similarity. Taxonomic ranks were assigned to OTU representative sequence by Ribosomal Database Project (RDP). At last, alpha diversity, beta diversity and the different species screening were analyzed based on OTU and taxonomic ranks. We used Monthur software [2] to observe the complexity of species based on α-diversity indexes of observed species, chao 1, ace, shannon, and simpson. The comparison of α-diversity indexes between Control, Model and RC group was performed by three different methods (metastats, kruskal.test and wilcox.test). Next, we used two-part model to identify the gut microbes associated with biochemical indicators and potential biomarkers in the cecum [3].

The two-part model contains a binary model and a quantitative model, respectively. The binary model explained the effect of the presence or absence of the gut microbiota on the obese rats’ biochemical indicators and potential biomarkers, and the quantitative model analyzed the effect of the abundance of the gut microbes on the obese rats’ biochemical indicators and potential biomarkers.

To further assess whether the effect came from the presence or absence or the abundance of the gut ﬂora or both, a combination of the binary and quantitative analysis was distinguished by a meta-analysis in which the *P*-value was gained employing an unweighted Z method. The equation of these three models were represented as below:

Binary Model: $y=\beta_{1}+e$


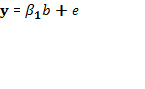


Quantitative Model: $y=\beta_{2}+e$


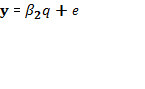


Unweighted Z method: $Z=\sum_{j=1}^{k} z_{i}/\surd k\sim N(0,1)$; $Z_{i}=\emptyset^{-1}(P_{i})$


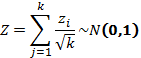


Where *y* represented the trait values (biochemical indicators and potential biomarkers) per individual after adjusting for body weight and length, *b* was the binary feature, *β_1_* and *β_2_* were the estimated effects of the binary and quantitative model, respectively, and *e* stood for the residuals. The *Z_i_* was the Z-transform test transforming the one tailed *P*-values, *P_i_*, was from each of *k* independent tests into a standard normal deviate. The *Z* was the sum of these *Z_i_* separated by the square root of the number of tests, *k* was a standard normal distribution [4].

The minimum of *P*-values came from the binary analysis, quantitative analysis and meta-analysis was set as the final association *P*-value. We carried out 1000 times permutation tests to manage the false discovery rate (FDR). The FDR ≤ 0.01 was installation as the significant threshold.

The FDR control: $FDR=N_{0}/N_{1}*1000\leq0.01$;

Where the *N_0_* was the average number of the investigated significance at a certain *P* cutoff in 1,000 permutations, *N_1_* was the probed positive in the real analysis.

**The total RNA in the liver determined using the TRIzol method and statistical analysis**

To identify the differential genes by RNA-Seq technology of liver tissue, we used three extreme individuals in each group on glucose and insulin level.

1）the total RNA of liver obtained by the TRIzol method

All the liver tissue samples were harvested from the rats on the ice box for RNA isolation within 15 min after euthanasia. Then, the samples were put into the sterile and frozen cryopreservation tubes with the liquid of RNA (Cwbiotech, China) and conserved in -80^0^C ultra freezer until RNA extraction. We used the TRIzol (Invitrogen, USA) to isolate the total RNA following the manufacture’s instruction.

2）statistical analysis

RNA-Seq is a simplified version of genome-wide transcripts. In short, mRNA was enriched by magnetic oligo (dT) beads (Invitrogen, USA). Then the mRNA was used as a template to synthesize cDNA used for PCR amplification and sequenced on a BGIseq-500 Sequencer (BGI, China). The raw data were analyzed by the BGI bioinformatics protocols for RNA-Seq. In briefly, all the raw data were firstly filtered to get the clean data. Then, the qualified data was mapping the rat reference transcript sets. To get the gene expression quantification, the Maximum likelihood abundance estimates was used by the RSEM tool [5]. The develop biomarker [6, 7] on RNA level was used to do condition specifically analysis. NOISeq method [8] could screen differentially expressed genes between two groups. To further understand the differentially expressed genes, KEGG [9] was used to perform pathway.

**References**

[1] D.W. Fadrosh, B. Ma, P. Gajer, N. Sengamalay, S. Ott, R.M. Brotman, J. Ravel, An improved dual-indexing approach for multiplexed 16S rRNA gene sequencing on the Illumina MiSeq platform, Microbiome, 2 (2014) 6.

[2] P.D. Schloss, S.L. Westcott, T. Ryabin, J.R. Hall, M. Hartmann, E.B. Hollister, R.A. Lesniewski, B.B. Oakley, D.H. Parks, C.J. Robinson, J.W. Sahl, B. Stres, G.G. Thallinger, D.J. Van Horn, C.F. Weber, Introducing mothur: open-source, platform-independent, community-supported software for describing and comparing microbial communities, Appl Environ Microbiol, 75 (2009) 7537-7541.

[3] J. Fu, M.J. Bonder, M.C. Cenit, E.F. Tigchelaar, A. Maatman, J.A. Dekens, E. Brandsma, J. Marczynska, F. Imhann, R.K. Weersma, L. Franke, T.W. Poon, R.J. Xavier, D. Gevers, M.H. Hofker, C. Wijmenga, A. Zhernakova, The Gut Microbiome Contributes to a Substantial Proportion of the Variation in Blood Lipids, Circ Res, 117 (2015) 817-824.

[4] M.C. Whitlock, Combining probability from independent tests: the weighted Z-method is superior to Fisher's approach, J Evol Biol, 18 (2005) 1368-1373.

[5] B. Li, C.N. Dewey, RSEM: accurate transcript quantification from RNA-Seq data with or without a reference genome, BMC Bioinformatics, 12 (2011) 323.

[6] M.D. Robinson, A. Oshlack, A scaling normalization method for differential expression analysis of RNA-seq data, Genome Biol, 11 (2010) R25.

[7] X. Yu, J. Lin, D.J. Zack, J. Qian, Computational analysis of tissue-specific combinatorial gene regulation: predicting interaction between transcription factors in human tissues, Nucleic Acids Res, 34 (2006) 4925-4936.

[8] S. Tarazona, F. Garcia-Alcalde, J. Dopazo, A. Ferrer, A. Conesa, Differential expression in RNA-seq: a matter of depth, Genome Res, 21 (2011) 2213-2223.

[9] M. Kanehisa, M. Araki, S. Goto, M. Hattori, M. Hirakawa, M. Itoh, T. Katayama, S. Kawashima, S. Okuda, T. Tokimatsu, Y. Yamanishi, KEGG for linking genomes to life and the environment, Nucleic Acids Res, 36 (2008) D480-484.

**Supplementary Tables and Figures**

Supplementary Table 1. The diet composition on normal chow diet and high-fat diet.

| Normal chow diet | | |  | High-fat diet | | |
| --- | --- | --- | --- | --- | --- | --- |
| Ingredient | gm% | kcal% |  | Ingredient | gm% | kcal% |
| Protein | 23.5 | 24 |  | Protein | 26.2 | 20 |
| Carbohydrate | 65.0 | 66 |  | Carbohydrate | 26.3 | 20 |
| Fat | 4.2 | 10 |  | Fat | 34.9 | 60 |

Supplementary Table 2. UPLC elution gradient for optimized UPLC-MS methods, ESI+. A is 99.9/0.1 water/formic acid and B is 99.9/0.1 acetonitrile/formic acid.

| Gradient | Time(min) | Flow(ml/min) | %A | %B | Cure |
| --- | --- | --- | --- | --- | --- |
| 1 | initial | 0.4 | 98 | 2 | initial |
| 2 | 2.0 | 0.4 | 98 | 2 | 6 |
| 3 | 4.0 | 0.4 | 85 | 15 | 6 |
| 4 | 6.0 | 0.4 | 55 | 45 | 6 |
| 5 | 15.0 | 0.4 | 30 | 70 | 6 |
| 6 | 19.0 | 0.4 | 1 | 99 | 6 |
| 7 | 20.0 | 0.4 | 1 | 99 | 6 |
| 8 | 23.0 | 0.4 | 98 | 2 | 1 |

Supplementary Table 3. Pre-administration: the of results the apparent indexes and serum biochemical indexes（$\overline{x}\pm s$）.

| Weeks | Weight | Length | LI | TC | TG | HDL-C | LDL-C | Insulin | HOMA-IR | GLU |
| --- | --- | --- | --- | --- | --- | --- | --- | --- | --- | --- |
| 0 |  |  |  |  |  |  |  |  |  |  |
| Control (n=8) | 182.97±11.66 | 18.96±0.81 | 299.57±9.05 | -- | -- | -- | -- | -- | -- | -- |
| Model (n=16) | 187.17±9.84 | 19.18±0.70 | 298.47±9.60 | -- | -- | -- | -- | -- | -- | -- |
| 1 |  |  |  |  |  |  |  |  |  |  |
| Control (n=8) | 244.68±11.72 | 20.66±0.47 | 302.75±7.33 | -- | -- | -- | -- | -- | -- | -- |
| Model (n=16) | 254.89±12.05**^*^** | 20.84±0.52 | 304.30±7.05 | -- | -- | -- | -- | -- | -- | -- |
| 2 |  |  |  |  |  |  |  |  |  |  |
| Control (n=8) | 279.42±10.15 | 22.36±0.74 | 292.60±8.09 | 1.59±0.23 | 0.98±0.30 | 0.45±0.04 | 0.95±0.16 | 2.08±0.11 | 0.44±0.05 | 4.79±0.41 |
| Model (n=16) | 315.40±18.08**^**^** | 22.69±0.52 | 299.91±6.39**^*^** | 1.84±0.25**^*^** | 1.24±0.46 | 0.55±0.26 | 1.04±0.33 | 2.15±0.28 | 0.50±0.11 | 5.27±0.90 |
| 3 |  |  |  |  |  |  |  |  |  |  |
| Control (n=8) | 300.54±8.36 | 23.20±0.45 | 288.80±6.43 | -- | -- | -- | -- | -- | -- | -- |
| Model (n=16) | 346.23±26.21**^**^** | 23.76±0.58**^*^** | 295.47±9.51 | -- | -- | -- | -- | -- | -- | -- |
| 4 |  |  |  |  |  |  |  |  |  |  |
| Control (n=8) | 321.30±12.93 | 24.03±0.40 | 285.05±5.28 | 1.53±0.19 | 0.91±0.21 | 0.46±0.05 | 0.89±0.16 | 1.86±0.20 | 0.32±0.07 | 3.89±0.37 |
| Model (n=16) | 381.16±29.26**^**^** | 24.55±0.44**^**^** | 295.17±8.04**^**^** | 1.79±0.30**^*^** | 1.48±0.54**^*^** | 0.48±0.06 | 1.01±0.23 | 2.14±0.28**^*^** | 0.45±0.08**^**^** | 4.74±0.66**^**^** |
| 5 |  |  |  |  |  |  |  | -- | -- |  |
| Control (n=8) | 348.41±28.48 | 24.64±0.50 | 285.52±10.35 | -- | -- | -- | -- | -- | -- | -- |
| Model (n=16) | 392.03±29.79**^**^** | 25.19±0.58**^*^** | 290.42±6.74 | -- | -- | -- | -- | -- | -- | -- |
| 6 |  |  |  |  |  |  |  | -- | -- |  |
| Control (n=8) | 366.44±32.75 | 25.43±0.51 | 281.21±6.06 | 2.16±0.21 | 1.25±0.17 | 1.30±0.20 | 0.61±0.14 | 2.96±0.28 | 0.52±0.08 | 3.99±0.77 |
| Model (n=16) | 413.08±32.04**^**^** | 25.61±0.55 | 290.63±5.89**^**^** | 2.48±0.33**^*^** | 1.46±0.42 | 1.64±0.29**^**^** | 0.56±0.11 | 3.01±0.36 | 0.65±0.13**^*^** | 4.92±1.09**^*^** |
| 7 |  |  |  |  |  |  |  |  |  |  |
| Control (n=8) | 389.27±32.55 | 25.46±0.57 | 286.56±5.40 | -- | -- | -- | -- | -- | -- | -- |
| Model (n=16) | 437.69±33.88**^**^** | 25.97±0.50**^*^** | 292.17±5.73**^*^** | -- | -- | -- | -- | -- | -- | -- |
| 8 |  |  |  |  |  |  |  |  |  |  |
| Control (n=8) | 405.60±32.47 | 25.75±0.41 | 287.28±6.02 | -- | -- | -- | -- | -- | -- | -- |
| Model (n=16) | 453.21±35.38**^**^** | 26.28±0.59**^*^** | 292.11±3.86**^**^** | -- | -- | -- | -- | -- | -- | -- |

Note：LI (Lee's index) and HOMA-IR calculated according to the following formula: power (weight,1/3) ×1000/length and glucose × insulin/22.5, respectively. Differences were assessed by one-way ANOWA, compared with the Control group, ^*^*P*<0.05, *^**^P*<0.01, --no data; TC (total cholesterol), TG (triglycerides), HDL-C (high-density lipoprotein cholesterol), LDL-C (low-density lipoprotein cholesterol).

Supplementary Table 4. After dosing: the of results the apparent indexes and serum biochemical indexes（$\overline{x}\pm s$）.

| Weeks | Weight | Length | LI | TC | TG | HDL-C | LDL-C | Insulin | HOMA-IR | GLU |
| --- | --- | --- | --- | --- | --- | --- | --- | --- | --- | --- |
| 1 |  |  |  |  |  |  |  |  |  |  |
| Control (n=8) | 422.72±27.38 | 26.53±0.38 | 282.81±3.57 | -- | -- | -- | -- | -- | -- | -- |
| Model (n=8) | 459.24±34.59 | 26.74±0.55 | 288.37±5.16 | -- | -- | -- | -- | -- | -- | -- |
| RC (n=8) | 479.56±52.63**^*^** | 26.89±0.69 | 290.77±8.29**^*^** | -- | -- | -- | -- | -- | -- | -- |
| 2 |  |  |  |  |  |  |  |  |  |  |
| Control (n=8) | 431.38±27.18 | 26.54±0.50 | 284.64±4.54 | 1.35±0.22 | 0.64±0.21 | 1.00±0.17 | 0.22±0.06 | 2.65±0.42 | 0.55±0.08 | 4.75±0.59 |
| Model (n=8) | 480.16±32.96 | 26.81±0.76 | 291.94±3.24**^*^** | 2.03±0.30**^**^** | 0.74±0.26 | 1.51±0.23**^**^** | 0.38±0.08 | 2.74±0.45 | 0.69±0.12**^*^** | 5.69±0.32**^**^** |
| RC (n=8) | 501.05±60.40**^**^** | 27.18±0.73 | 291.78±6.14**^*^** | 2.07±0.31**^**^** | 0.84±0.39 | 1.48±0.26**^**^** | 0.42±0.13**^**^** | 2.48±0.36 | 0.56±0.08**^#^** | 5.12±0.50 |
| 3 |  |  |  |  |  |  |  |  |  |  |
| Control (n=8) | 440.61±27.10 | 26.39±0.62 | 288.37±7.38 | -- | -- | -- | -- | -- | -- | -- |
| Model (n=8) | 474.41±43.10 | 27.04±0.69 | 288.23±7.18 | -- | -- | -- | -- | -- | -- | -- |
| RC (n=8) | 513.02±65.28**^*^** | 27.39±0.64**^*^** | 291.75±7.83 | -- | -- | -- | -- | -- | -- | -- |
| 4 |  |  |  |  |  |  |  |  |  |  |
| Control (n=8) | 459.51±17.87 | 27.15±0.69 | 284.30±6.11 | 1.50±0.17 | 0.97±0.08 | 1.14±0.13 | 0.16±0.06 | 2.22±0.22 | 0.67±0.08 | 6.77±0.75 |
| Model (n=8) | 479.91±41.28 | 27.19±0.65 | 287.85±8.72 | 1.92±0.15**^**^** | 1.11±0.24 | 1.54±0.17**^*^** | 0.16±0.04 | 2.27±0.23 | 0.87±0.17**^*^** | 8.61±1.10**^**^** |
| RC (n=8) | 514.59±59.28 | 27.61±0.58 | 289.80±7.74 | 1.94±0.24**^**^** | 1.17±0.36 | 1.53±0.26**^*^** | 0.18±0.16 | 2.31±0.31 | 0.60±0.09**^##^** | 5.90±0.57**^##^** |

Note：LI (Lee's index) and HOMA-IR calculated according to the following formula: power (weight,1/3) ×1000/length and glucose × insulin/22.5, respectively. Differences were assessed by one-way ANOWA, compared with the Control group, **P*<0.05, ***P*<0.01, compared with the Model group, ^#^*P*<0.05, ^##^*P*<0.01, --no data, TC (total cholesterol), TG (triglycerides), HDL-C (high-density lipoprotein cholesterol), LDL-C (low-density lipoprotein cholesterol).

Supplementary Table 5. After dosing: the of results amount of food ingested.

| Weeks | Average weight (g) | Amount of food ingested (g) | Average amount of food ingested (g/weight) |
| --- | --- | --- | --- |
| 1 |  |  |  |
| Control (n=8) | 422.72±27.38 | 169.09 | 0.05 |
| Model (n=8) | 459.24±34.59 | 183.70 | 0.05 |
| RC (n=8) | 479.56±52.63 | 191.82 | 0.05 |
| 2 |  |  |  |
| Control (n=8) | 431.38±27.18 | 172.55 | 0.05 |
| Model (n=8) | 480.16±32.96 | 192.06 | 0.05 |
| RC (n=8) | 501.05±60.40 | 200.42 | 0.05 |
| 3 |  |  |  |
| Control (n=8) | 440.61±27.10 | 246.74 | 0.07 |
| Model (n=8) | 474.41±43.10 | 265.67 | 0.07 |
| RC (n=8) | 513.02±65.28 | 287.29 | 0.07 |
| 4 |  |  |  |
| Control (n=8) | 459.51±17.87 | 220.56 | 0.06 |
| Model (n=8) | 479.91±41.28 | 230.36 | 0.06 |
| RC (n=8) | 514.59±59.28 | 247.00 | 0.06 |

Supplementary Table 6. The differences metabolites and association pathways of the Control and Model groups. Please see the Supplementary Table 6.xlsx.

Supplementary Table 7. The differences metabolites and association pathways of the RC and Model groups. Please see the Supplementary Table 7.xlsx.


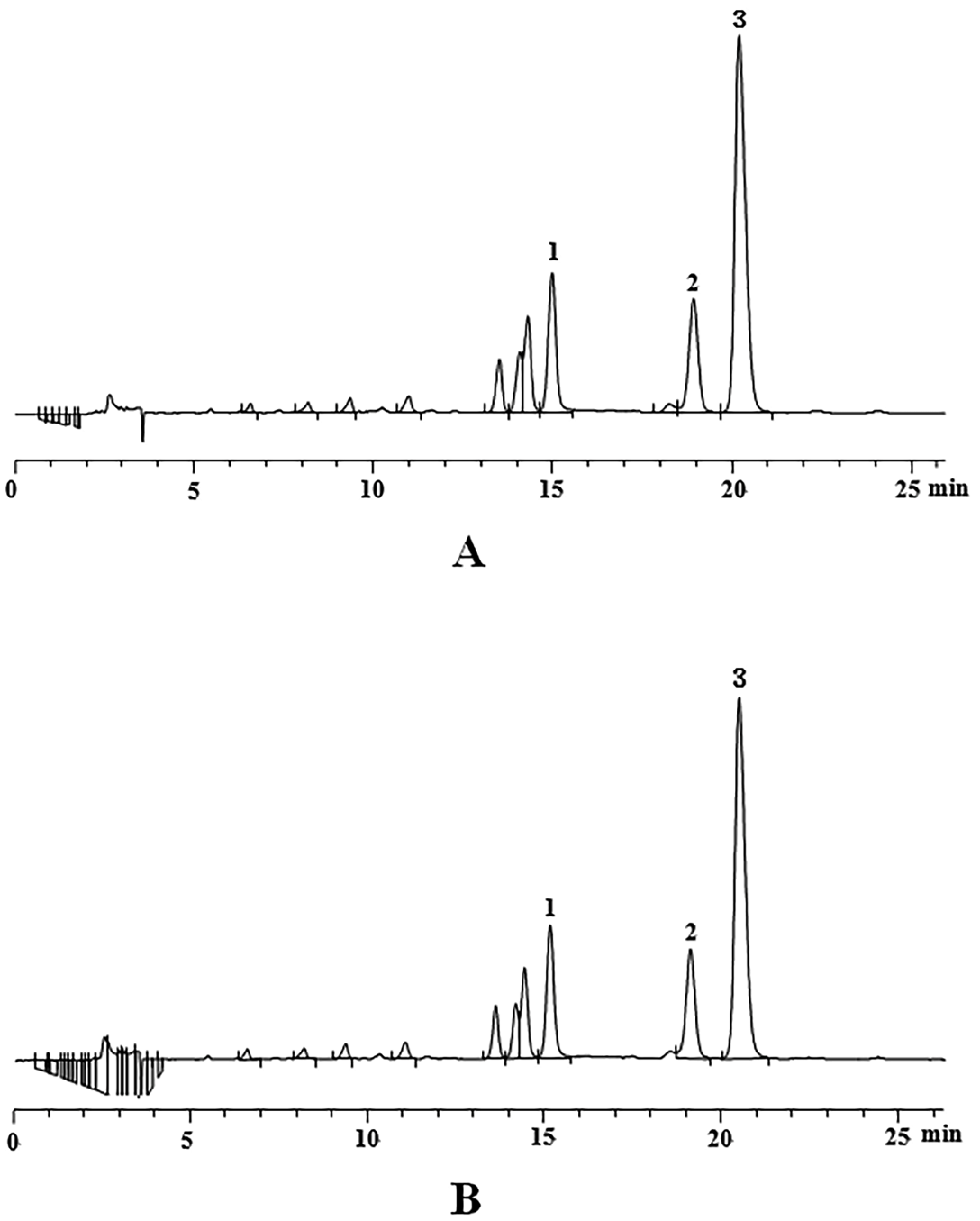


Supplementary Figure 1. HPLC fingerprint of the RC decoction. (A) HPLC-UV chromatogram of Batch A. (B) HPLC-UV chromatogram of Batch B. Peaks were detected at 345nm (1, coptisine; 2, palmatine; 3, berberine hydrochloride).


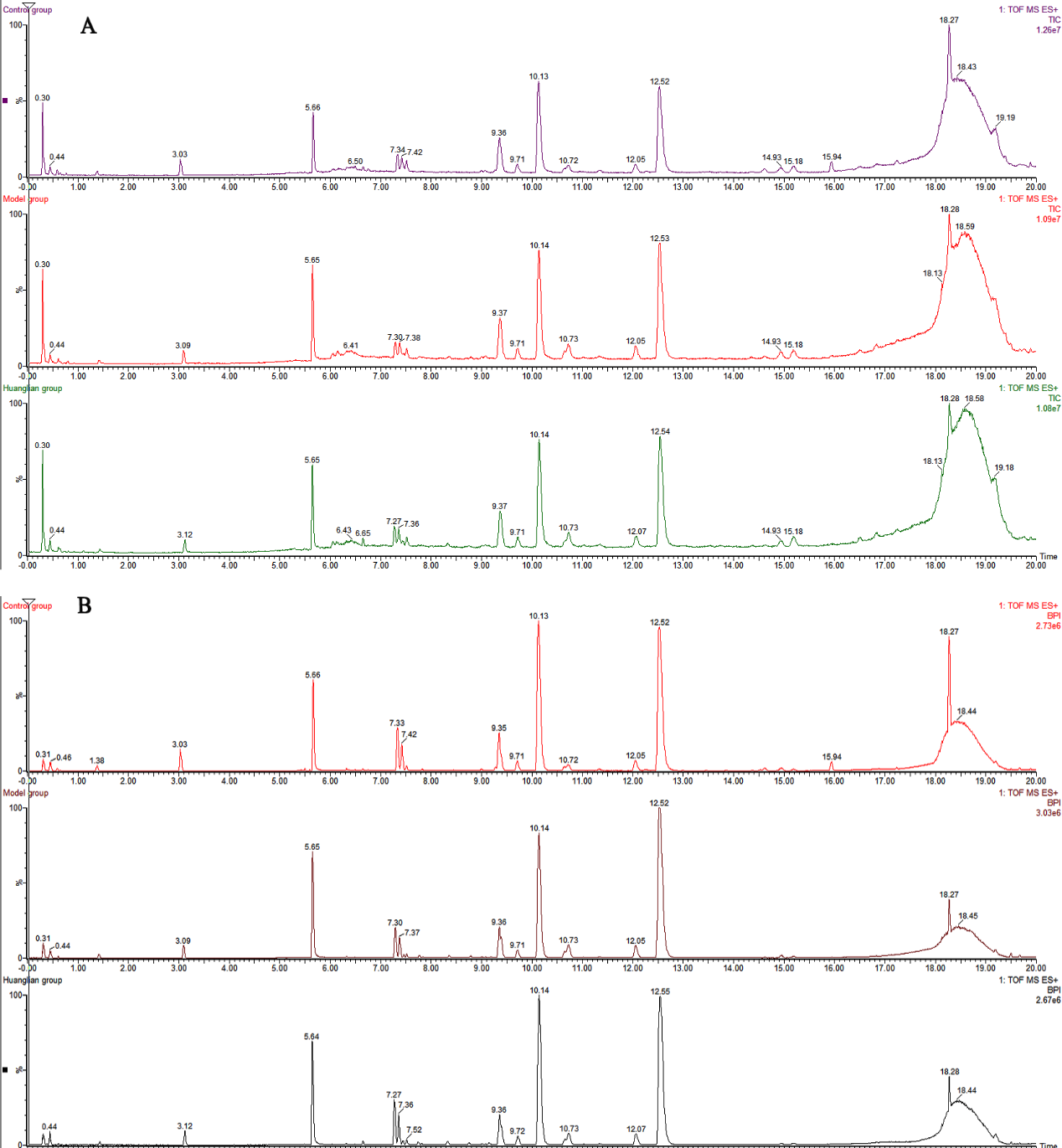


Supplementary Figure 2. The chromatograms of the serum sample of the rats in positive mode. (A) Typical TIC chromatogram obtained from the same serum sample of the rats with positive mode. (B) Typical BPI chromatogram obtained from the same serum sample of the rats with positive mode.


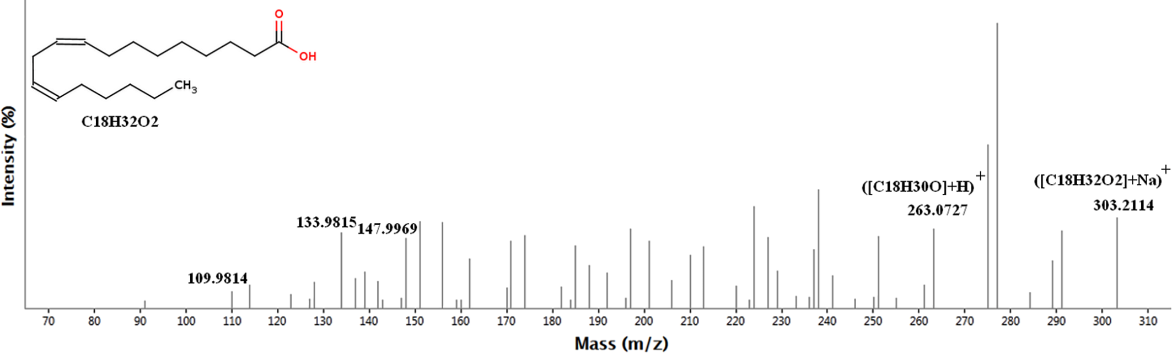


Supplementary Figure 3. Identification of a potential marker. MS/MS spectrum; the collision energy was 20eV~30eV.


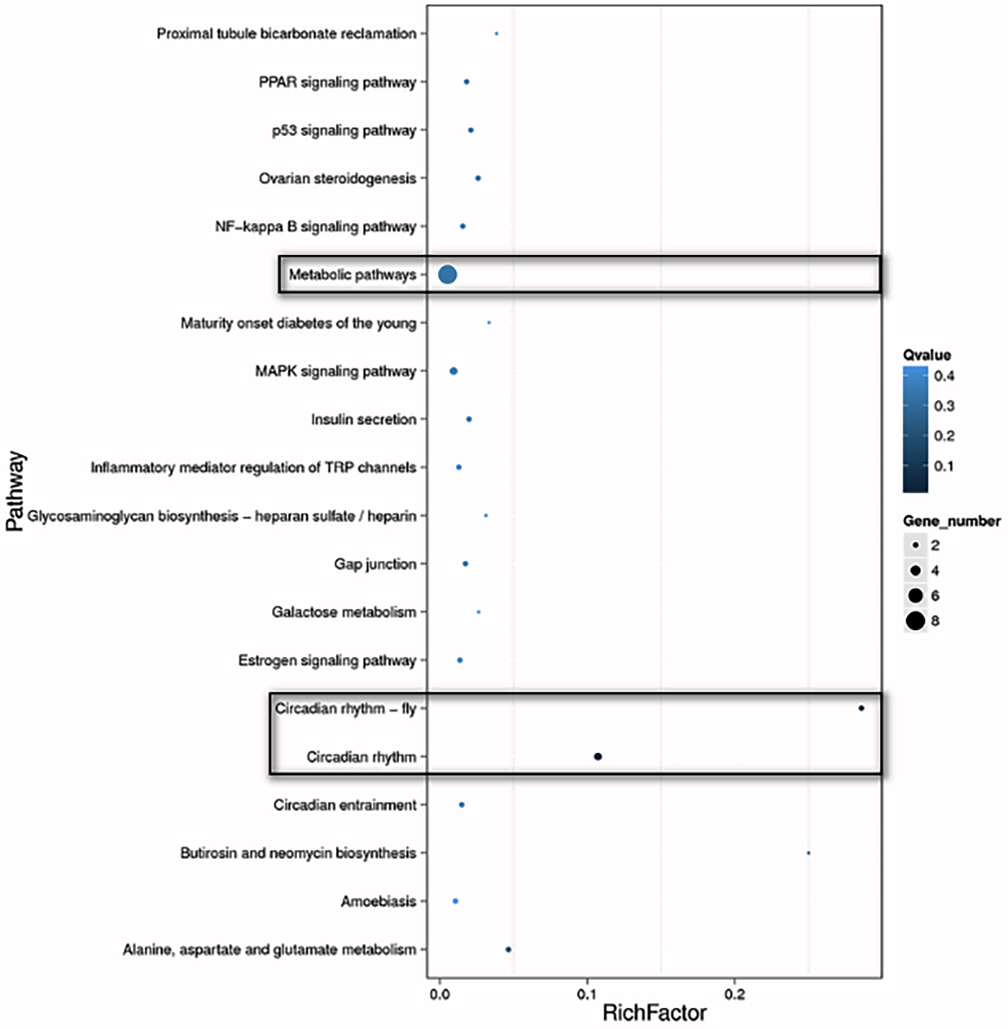


Supplementary Figure 4. The pathway enrichment of differentially expressed genes of the liver tissue.


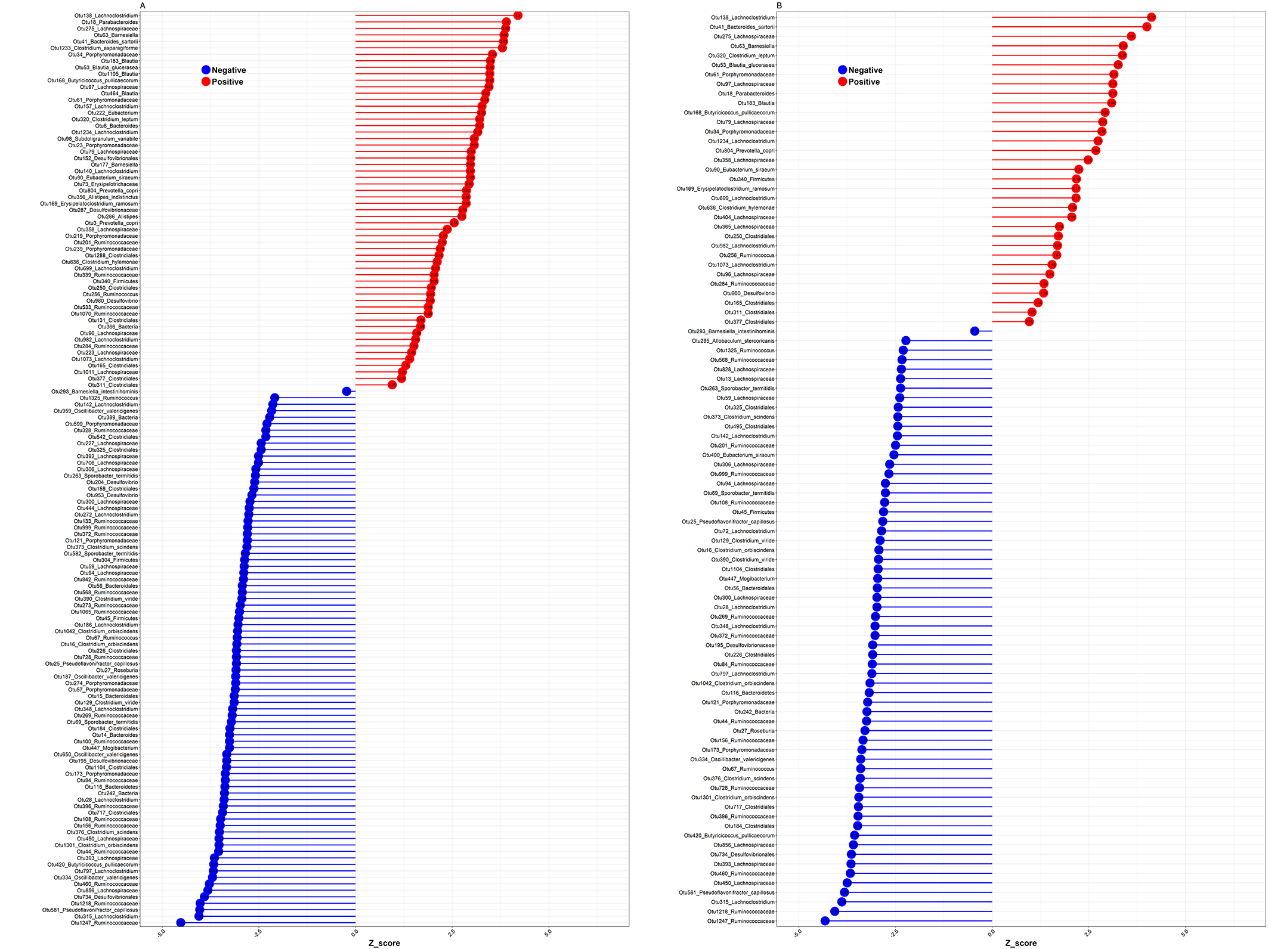


Supplementary Figure 5. The relationship between the biochemical indicators and gut microbiota. (A) The results of TC. (B) The results of HDL-C.
